# Team climate mediates the effect of diversity on environmental science team satisfaction and data sharing

**DOI:** 10.1371/journal.pone.0219196

**Published:** 2019-07-18

**Authors:** Isis H. Settles, Sheila T. Brassel, Patricia A. Soranno, Kendra Spence Cheruvelil, Georgina M. Montgomery, Kevin C. Elliott

**Affiliations:** 1 Department of Psychology, University of Michigan, Ann Arbor, MI, United States of America; 2 Department of Afroamerican and African Studies, University of Michigan, Ann Arbor, MI, United States of America; 3 Department of Fisheries and Wildlife, Michigan State University, East Lansing, MI, United States of America; 4 Lyman Briggs College, Michigan State University, East Lansing, MI, United States of America; 5 Department of History, Michigan State University, East Lansing, MI, United States of America; 6 Department of Philosophy, Michigan State University, East Lansing, MI, United States of America; University of Maryland Baltimore County, UNITED STATES

## Abstract

Scientific research—especially high-impact research—is increasingly being performed in teams that are interdisciplinary and demographically diverse. Nevertheless, very little research has investigated how the climate on these diverse science teams affects data sharing or the experiences of their members. To address these gaps, we conducted a quantitative study of 266 scientists from 105 NSF-funded interdisciplinary environmental science teams. We examined how team climate mediates the associations between team diversity and three outcomes: satisfaction with the team, satisfaction with authorship practices, and perceptions of the frequency of data sharing. Using path analyses, we found that individuals from underrepresented groups perceived team climate more negatively, which was associated with lower satisfaction with the team and more negative perceptions of authorship practices and data sharing on the team. However, individuals on teams with more demographic diversity reported a more positive climate than those on teams with less demographic diversity. These results highlight the importance of team climate, the value of diverse teams for team climate, and barriers to the full inclusion and support of individuals from underrepresented groups in interdisciplinary science teams.

## Introduction

Several trends are transforming contemporary scientific practices. In most disciplines, scientific research is increasingly being conducted in teams [1–3], and these teams are becoming increasingly interdisciplinary in order to tackle grand challenges that require multiple disciplinary perspectives [4,5]. The scientific community is also striving to become more demographically diverse and to promote the advancement of groups that have been underrepresented in the sciences [6,7].

However, creating successful teams that are demographically and scientifically diverse is not a simple matter of recruiting more individuals from underrepresented groups and combining team members from a variety of disciplinary backgrounds. Diverse teams can struggle with allocation of credit, differences in perspectives, and unequal power dynamics. For example, women and those from “soft sciences” (e.g., sociology) can be less credited or valued than men or those from “hard sciences” (e.g., physics; [8–10]). Philosophical, methodological, and conceptual differences that result from disciplinary diversity can complicate team collaboration [4,5,11]. Finally, power dynamics can be difficult in demographically diverse teams, with individuals from some groups feeling unable to influence team practices and decisions [12]. For the sciences to effectively transition to a more diverse team-based enterprise, contemporary science teams must address these challenges.

We propose that *team climate* is a critical factor for addressing these challenges and promoting the success of diverse science teams. Team climate is the perceived set of norms, attitudes, and expectations on a team [13]. Research indicates that climate is related to individual job attitudes such as organizational commitment and turnover intentions, as well as to job performance [14–16]. Climate is also related to positive team performance [17,18]. However, surprisingly few studies have investigated the relationship between climate and diversity specifically within science teams [5]. In one of the few studies of diversity and team climate in the science team context, Li et al. [19] found that cultural diversity was related to greater creativity on engineering teams through the mediating role of information sharing, but that result was only for teams with a climate of inclusion (i.e., equitable employment practices, integration of differences, and collective decision making). To better support science teams with increased demographic and disciplinary diversity, more studies are needed to determine how individual and team diversity are related to climate, perceptions of team functioning, and team satisfaction.

**Fig 1:**
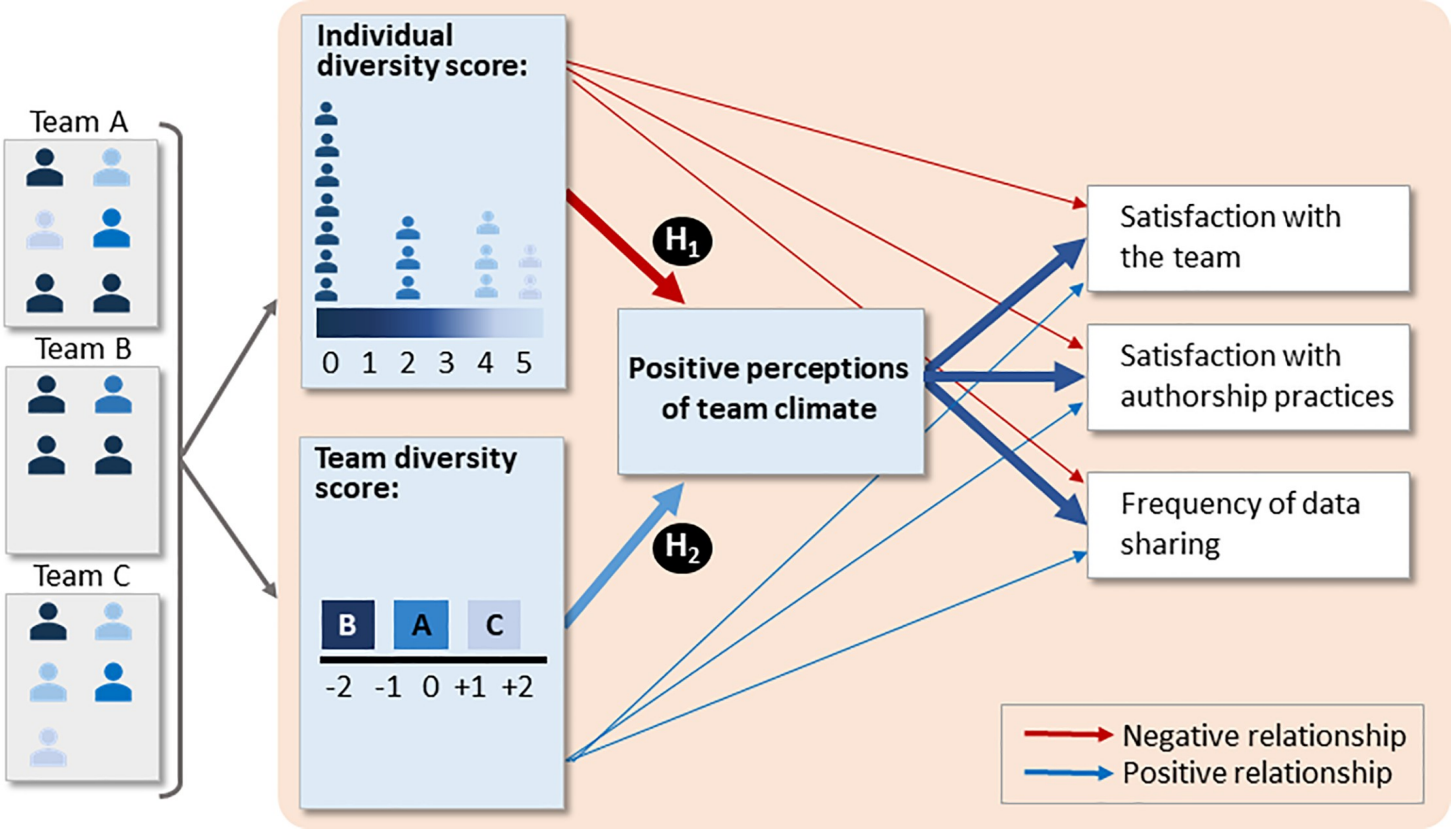
Conceptual model showing how individual and team diversity likely affects environmental science team outcomes, as mediated by climate perceptions. H_1_ and H_2_ are hypotheses about the relationships between demographic and scientific diversity, team climate, and team outcomes (see text for details). Line thickness indicates hypothesized strength of relationships.

**Fig 2:**
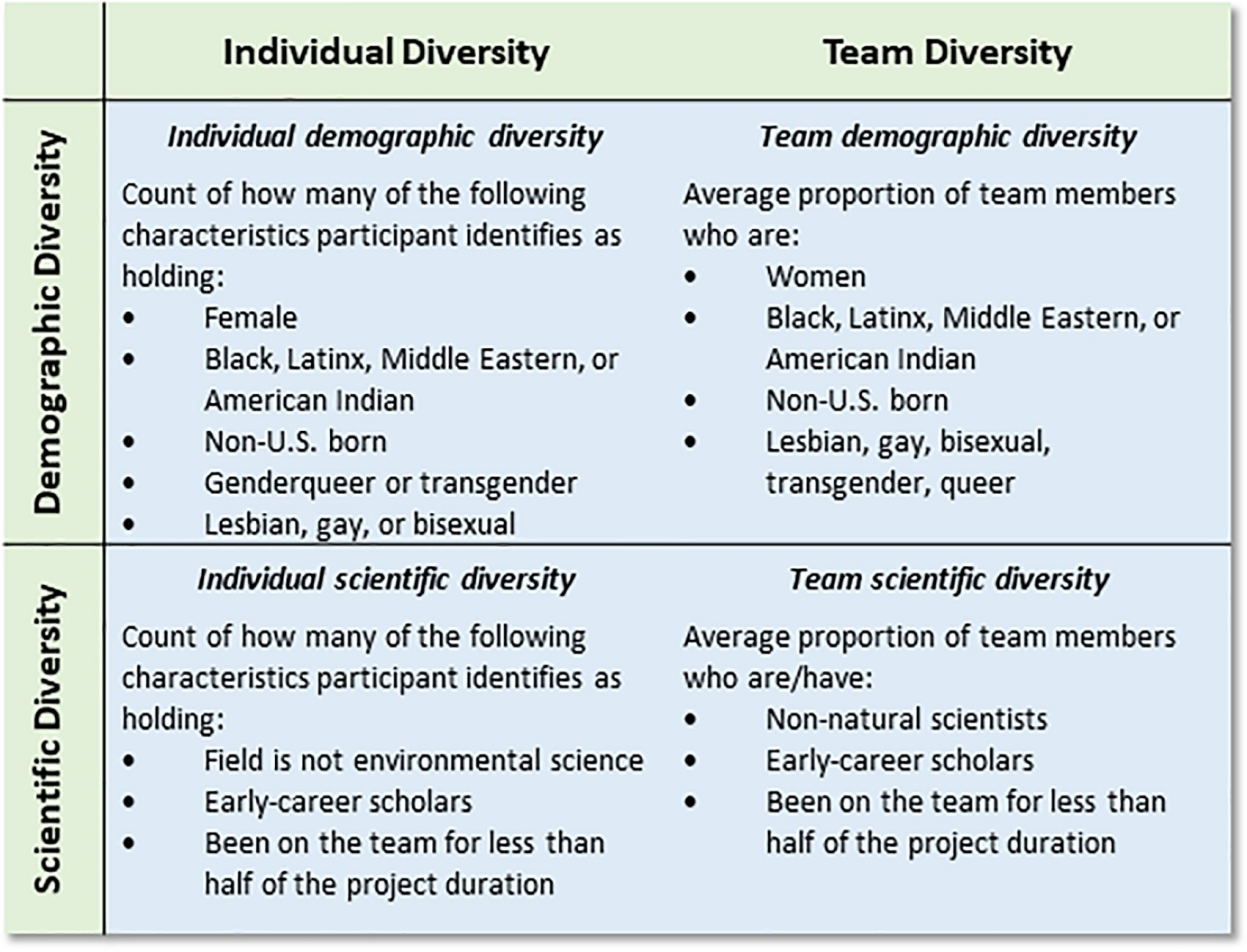
Defining diversity composite variables, both at the individual and team levels based on demographic and scientific characteristics.

To fill this gap, our study tests a conceptual framework that describes how diversity, climate, and team outcomes are related to each other (Fig 1) in a sample of 266 participants from 105 NSF-funded interdisciplinary environmental science teams. We studied the associations between two forms of individual and team diversity (demographic and scientific; see Fig 2) and team members’ satisfaction with their teams, their satisfaction with authorship practices, and perceptions of the frequency of team data sharing. These outcomes are important because diverse, interdisciplinary science teams are less likely to function successfully and to retain members of underrepresented groups if team members are not satisfied and if they do not perceive effective and fair implementation of important team practices such as authorship and data sharing. By measuring diversity in terms of demographic and scientific composites that simultaneously account for multiple underrepresented identities, our study builds on previous work that has focused on single dimensions of diversity, such as gender or race [5], which does not accurately reflect people’s lived experience.

**Fig 3:**
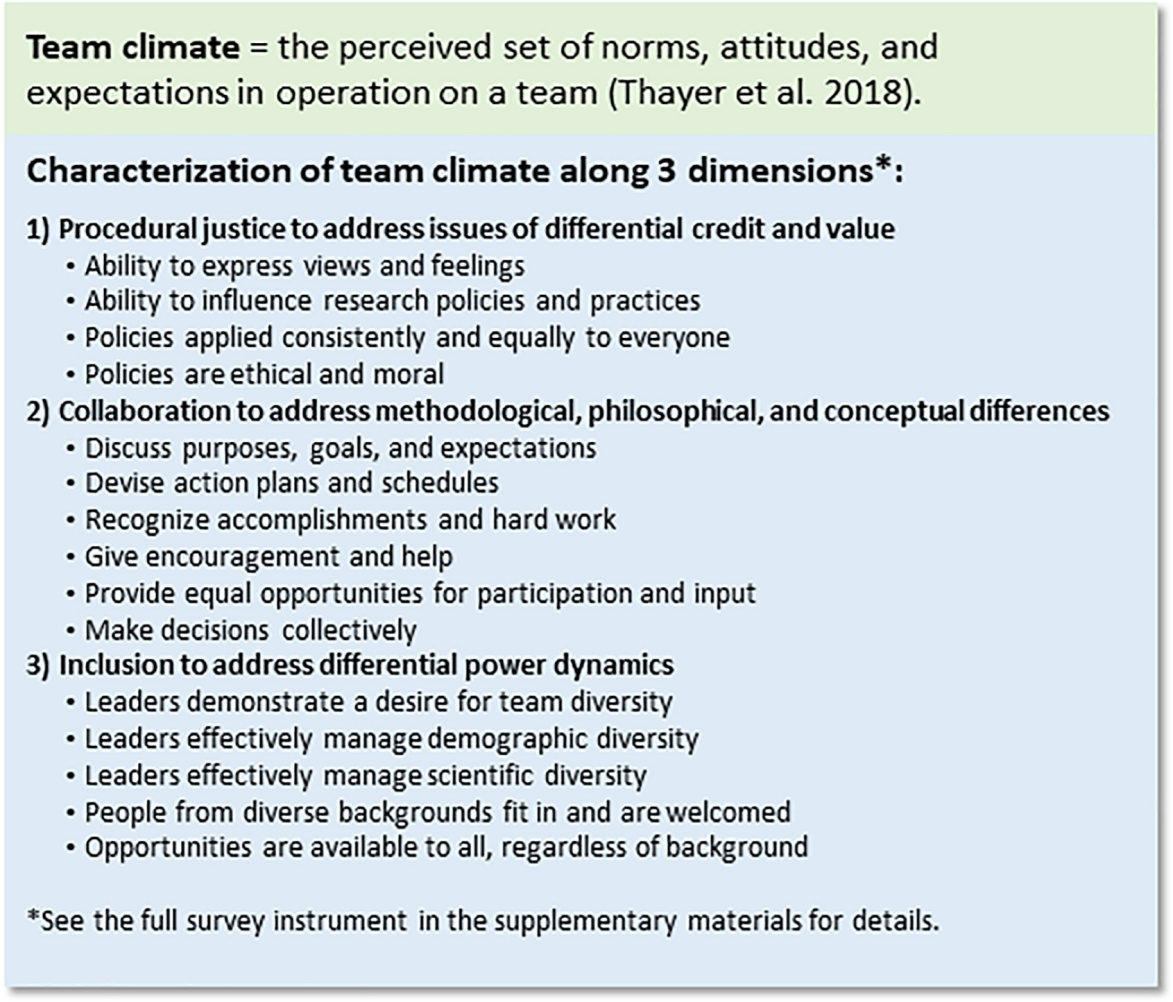
Team climate definition and characterization along the three dimensions of procedural justice, collaboration, and inclusion.

We included team climate as a mediator of the associations between diversity and outcomes, and we measured it by assessing individuals’ perceptions of procedural justice, collaboration, and inclusion on their teams (see Fig 3). These dimensions of climate are likely to help teams address challenges associated with allocation of credit, differences in perspectives, and unequal power dynamics [20–22].

We hypothesized (Fig 1) that: (H_1_) individuals with more underrepresented demographic and scientific characteristics compared to their counterparts will be less satisfied with their teams, less satisfied with authorship practices specifically, and perceive data sharing to occur less often, and these associations will be mediated by more negative perceptions of team climate; and (H_2_) regardless of one’s own demographic and scientific characteristics, individuals who are on teams with more demographic and scientific diversity will be more satisfied with their teams, more satisfied with authorship practices specifically, and perceive data sharing to occur more often compared to individuals on teams that have less demographic and scientific diversity, and these associations will be mediated by more positive perceptions of team climate. To test these hypotheses, we conducted path analyses to understand how team satisfaction and team practices were affected by diversity composites, and how team climate mediated these associations.

## Methods

### Participants and survey procedures

Potential participants and teams were identified using the National Science Foundation (NSF) database of awards for three interdisciplinary environmental science funding programs. (We do not provide program names to reduce risk of participants’ identification.) The NSF database reports contact information for project Principal Investigators (PIs) and Co-Principal Investigators (Co-PIs). To recruit participants who held other roles on these projects (e.g., graduate student research assistants, post-docs, technicians), we emailed the PIs and requested that they provide contact information for all team members. During the summer of 2017, we invited a total of 1,727 individuals from 229 interdisciplinary research teams via email to participate in an online survey using the Qualtrics survey platform. To increase survey responses, participants had the opportunity to win one of five $100 Amazon gift cards, and we sent two follow-up reminder emails to non-respondents. The survey contained our NSF (NSF-14546) and IRB (HUM00128956) identification numbers, contact information for our project Principal Investigator and Research Coordinator, and instructions that participants could skip any questions that they preferred not to answer.

Our final sample contained 266 participants from 105 NSF-funded research teams (response rate = 15.4% of participants, 45.9% of teams). Participants had an average age of 46.56 (*SD* = 13.15) years. See Table 1 for participants’ sex, race/ethnicity, sexual orientation and gender identity, nationality, academic disciplines, career status, and duration of involvement with their NSF research projects; see Table 2 for the demographic and scientific composition of participants’ teams. Correlations among all study variables are presented in Table 3. All data included in this study, except for demographic information that could be used to identify participants, are available in a public archive [23].

**Table 1:** Demographic and scientific characteristics of survey respondents.

Variable	Characteristic	*n* (%)
*Demographic characteristics of participants*		
Sex	Male	153 (57.5%)
	Female	109 (41.0%)
Race	White	202 (76.0%)
	Hispanic/Latinx	27 (10.2%)
	Asian/Asian American/Pacific Islander	21 (7.9%)
	Other ^a^	7 (2.6%)
Sexual Orientation & Gender Identity	Straight/Heterosexual	224 (84.2%)
	LGBTQ^a^	28 (10.5%)
Birth Country	United States	195 (73.3%)
	Outside the United States	68 (25.6%)
*Scientific characteristics of participants*		
Academic Discipline^b^	Natural Sciences	226 (85.0%)
	Environmental Sciences	207 (77.8%)
	Social Sciences	59 (22.2%)
	Mathematics and Computer Sciences	20 (7.5%)
	Engineering	12 (4.5%)
	Humanities	8 (3.0%)
Career Status	Post-Undergrad Research Assistant/Technician	19 (7.1%)
	Graduate Student	52 (19.5%)
	Post-Doc	37 (13.9%)
	Tenure-track Assistant Professor or Assistant Scientist	19 (7.1%)
	Fixed-term Assistant or Associate Professor	15 (5.6%)
	Tenured Associate Professor or Associate Scientist	34 (12.8%)
	Tenured Full Professor, Fixed-term Full Professor, or Senior Scientist	89 (33.5%)
Project Tenure	Half or more of the project duration	235 (88.3%)
	Less than half of the project duration	29 (10.9%)

*Note*. *N* = 266. Not all participants provided all their demographic and scientific information; thus, each set of characteristics does not sum to 266.

^a^ To protect participant anonymity, groups with fewer than 10 members are not reported separately. The race category “Other” presents the aggregate proportion of participants who identified as Black/African American, Middle Eastern, or Native American/First Nations/American Indian. We have also aggregated participants who identified as lesbian, gay, bisexual, transgender, genderqueer, queer, asexual, or pansexual (LGBTQ).

^b^ Participants could affiliate with multiple academic disciplines.

**Table 2:** Demographic and scientific composition of the teams of the survey respondents.

Variable	Characteristic	Mean proportion of team, *n* (% of team)
*Demographic composition of the team*		
Sex	Male	7.62 (59.2%)
	Female	5.13 (39.9%)
Race	White	10.05 (78.1%)
	Hispanic/Latinx	0.92 (7.2%)
	Asian/Asian American/Pacific Islander	1.08 (8.4%)
	Black	0.21 (1.6%)
	Middle Eastern	0.13 (1.0%)
	Native American	0.18 (1.4%)
Sexual Orientation & Gender Identity^a^	Lesbian, gay, bisexual, and queer-identified	*Mode* = 2 (Mostly Straight/Heterosexual)
Nationality^b^	From countries outside the U.S.	*Mode* = 2 (Mostly from the U.S.)
*Scientific composition of the team*		
Academic Discipline	Natural Sciences	8.40 (65.3%)
	Social Sciences	3.20 (24.9%)
	Mathematics and Computer Sciences	1.27 (9.9%)
	Engineering	1.23 (9.6%)
	Humanities	.55 (4.3%)
Career Status	Lead Principal Investigator	1.70 (13.2%)
	Co-Principal Investigator	4.40 (34.2%)
	All other team members	8.03 (62.4%)
Previous Collaboration^c^	Proportion previously collaborated	*Mode* = 2 (21–40%) and 3 (41%-60%)

*Note*. Because team composition for Sexual Orientation, Citizenship & Nationality, and Previous Collaboration were measured on 5-point Likert-type scales rather than numerically, we present their modal responses in the table.

^a^ Sexual Orientation was measured on a scale from 1 (*all straight/heterosexual*) to 5 (*all lesbian*, *gay*, *bisexual*, *and queer*).

^b^ Nationality was measured on a scale from 1 (*all from the U*.*S*.) to 5 (*all not from the U*.*S*.).

^c^ Previous Collaboration was measured on a scale from 1 (*0–20%*) to 5 (*81–100%*) and had two modal values of 2 (*21–40%*) and 3 (*41–60%*); thus, both modes are presented in the table.

**Table 3:** Correlations, means, standard deviations, and ranges for participants’ responses to all study variables.

	1.	2.	3.	4.	5.	6.	7.	8.
1. Individual Demographic Diversity	–							
2. Team Demographic Diversity	.26***	–						
3. Individual Scientific Diversity	.20***	-.04	–					
4. Team Scientific Diversity	.03	.16**	.06	–				
5. Team Climate	-.13*	.13*	-.22***	.04	–			
6. Satisfaction with the team	-.03	.11	-.16*	.00	.76***	–		
7. Satisfaction with authorship practices	-.13*	.07	-.30***	.02	.65***	.56***	–	
8. Frequency of data Sharing	-.07	.06	-.07	.00	.44***	.32***	.51**	–
*M* (*SD*)	.89 (.87)	-.01 (.57)	.86 (.71)	-.02 (.62)	4.10 (.66)	4.47 (.88)	4.34 (.73)	4.43 (.76)
*Range*	0–3	-1.18–1.68	0–3	-1.87–1.36	1.14–5.00	1.00–5.00	1.00–5.00	1.50–5.00

*Note*. *Ns* = 232–266. All variables are coded such that higher numbers indicate higher scores on that variable.

*p < .05

**p < .01

***p ≤ .001

### Characterizing diversity

We used the survey responses to calculate four composite predictor variables characterizing diversity in our models. We computed measures of diversity at both the *individual* and the *team* level, as well as to characterize both *demographic* and *scientific* diversity (See S1 Table for additional details on the diversity composite variables).

*Individual demographic diversity* was a sum of the number of dimensions (ranging from 0 to 5) along which participants contributed to their team’s demographic diversity in terms of sex, race, sexual orientation, gender identity, and nationality (see Fig 2 for the specific groups coded as contributing diversity in each category). Groups that are underrepresented in the academy relative to their prevalence in the United States were coded as contributing diversity to their teams. Note that for race, we did not count being Asian as contributing to diversity because they are not underrepresented in the academy [24,25], and racial stereotypes portray Asians as intelligent, educated, and hard-working–characteristics that are not as readily attributed to other racial minority groups [26]. As a result, Asians in the academy may have qualitatively different experiences from those of racial minorities who are underrepresented in the academy [27].

*Team demographic diversity* represents participants’ teams’ demographic diversity in terms of sex, race, sexual orientation and gender identification, and nationality. Using participant reports of the gender and race of team members, and total numbers of team members, we calculated the proportion of team members providing gender and racial diversity (see Fig 2 for the specific groups coded as contributing diversity in each category). As with individual demographic diversity, Asians were not included when calculating team racial diversity. For sexual orientation and nationality, participants indicated, to the best of their knowledge, the makeup of their team on a 5-point Likert-type scale; sexual orientation ranged from 1 (*all straight/heterosexual*) to 5 (*all lesbian*, *gay*, *bisexual*, *and queer-identified*), and nationality ranged from 1 (*all from the U*.*S*.) to 5 (*all not from the U*.*S*.). “Don’t know” and “Prefer not to answer” response options were available for these questions, and these responses were excluded from the measure. Because these variables had different scales, participants’ scores on each variable were standardized and then averaged, so that higher scores indicated more team demographic diversity on these four dimensions.

*Individual scientific diversity* was computed as the sum of the number of dimensions (ranging from 0 to 3) along which participants contributed to their team’s scientific diversity in terms of academic discipline, career status, and how long they had been involved with their team’s project. Groups with less status on an environmental science team were coded as contributing diversity to their teams (see Fig 2 for the specific groups coded as contributing diversity in each category).

*Team scientific diversity* represented individuals’ reports of the scientific diversity on their teams in terms of career status, discipline, and previous collaboration. Participants reported the number of team members in various career positions and in different disciplines; using their reports of the total number of team members, we calculated the proportion of team members providing diversity in each category. For previous collaboration, participants indicated the proportion of team members who had previously collaborated on a scale from 1 = 0–20%, 2 = 21–40%, 3 = 41–60%, 4 = 61–80%, 5 = 81–100% (see Fig 2 for the specific groups coded as contributing diversity in each category). These three variables were standardized and averaged so that higher scores indicated more team scientific diversity.

### Characterizing team climate

We assessed participants’ perceived climate on their team via measures of *procedural justice*, *team collaboration*, and how much the *team values inclusion*. For all three of these measures, we adapted questions from published scales (see S2 Table for all scale items). For each scale, we computed a mean score such that higher values reflect a more positive climate.

To measure *procedural justice*, we adapted four items from the Procedural Justice subscale of Colquitt’s [21] Organizational Justice Scale. Participants responded to items (e.g., “Have you had the ability to influence your NSF team’s policies and/or practices related to conducting and publishing research?”; *M* = 4.13, *SD* = .82; Cronbach’s alpha = .84) on a scale from 1 (*not at all*) to 5 (*almost always*).

To measure *team collaboration*, we adapted six items from Carson et al.’s [20] assessment of Internal Team Environment for Shared Leadership. Participants responded to items (e.g., “The members of the team spend time discussing our team's purpose, goals, and expectations for the project”; *M* = 4.02, *SD* = .67; Cronbach’s alpha = .87) on a scale from 1 (*strongly disagree*) to 5 (*strongly agree*).

To measure *team value of inclusion*, we adapted six items from Pugh et al.’s [22] Diversity Climate measure. Participants responded to items (e.g., “Our team makes it easy for people from diverse backgrounds to fit in and be accepted”; *M* = 4.17, *SD* = .72; Cronbach’s alpha = .88) on a scale from 1 (*strongly disagree*) to 5 (*strongly agree*).

In order to measure *team climate*, we combined these three mean scale scores such that higher scores indicated more positive climate on the team. Cronbach’s alpha for the composite was 0.93 for our sample. The correlations between all subscales were significant at *p* < .001 (S1 Table).

### Characterizing team outcomes

We used the survey responses to calculate three response variables for our models that characterize individuals’ experiences on their interdisciplinary science teams. We measured individuals’ *satisfaction with their team*, their *satisfaction with its authorship practices*, and their perceptions of its *data sharing practices*. We based the survey questions on prior qualitative interviews of the population [28]. To measure the extent to which participants were *satisfied* with their experiences on their interdisciplinary science teams, participants responded to the question “Overall, how satisfied are you with your experiences on your interdisciplinary NSF-funded science team?” on a scale from 1 (*not at all satisfied*) to 5 (*very satisfied*).

To measure participants’ satisfaction with their interdisciplinary science teams’ *authorship practices*, we asked them to answer three questions about authorship credit: “To what extent do you think that you personally received appropriate credit (in terms of being included as an author or not) on the papers published by your team?” 1 (*inappropriate*) to 5 (*appropriate*); “In your personal opinion, to what extent do you think your interdisciplinary NSF-funded science team is typically fair in deciding who to include as authors on papers?” 1 (*not at all fair*) to 5 (*extremely fair*); and “How often do you think your interdisciplinary NSF-funded science team has excluded people from being authors even though they contributed sufficiently to the paper?” 1 (*never*) to 5 (*always*). The exclusion item was reverse-scored and then the three questions were averaged such that higher scores indicate more satisfaction with team authorship practices. The correlations between all items were significant with *p* < .001 and Cronbach’s alpha was 0.75 for our sample.

To measure the *data sharing practices*, we asked participants to indicate how often their team shared data within sub-teams (i.e., a smaller group of team members working on a specific task within the larger research team) and with the entire team using a 5-point Likert-type scale of 1 (*never*) to 5 (*always*). The correlation between data sharing within sub-teams and with the entire team was significant *r* = .51, *p* < .001. Scores for the two items were averaged and higher scores indicate more data sharing within the team.

## Results

To test our hypotheses, we used the conceptual model described in Fig 1 and conducted two path analyses, one with each set of diversity composites (i.e., individual and team *demographic* diversity or individual and team *scientific* diversity) as the predictor variables. In all analyses, the mediator was team climate and the outcomes were satisfaction with the team, satisfaction with authorship practices, and data sharing practices. Path analysis, an extension of multiple regression, tests the strength of relationships among variables. Because it can test a hypothesized model with multiple independent and dependent variables, and mediating (i.e., indirect) effects, it is appropriate for our study [29]. All analysis used MPLUS Version 8 [30].

### Demographic diversity and team climate

**Fig 4:**
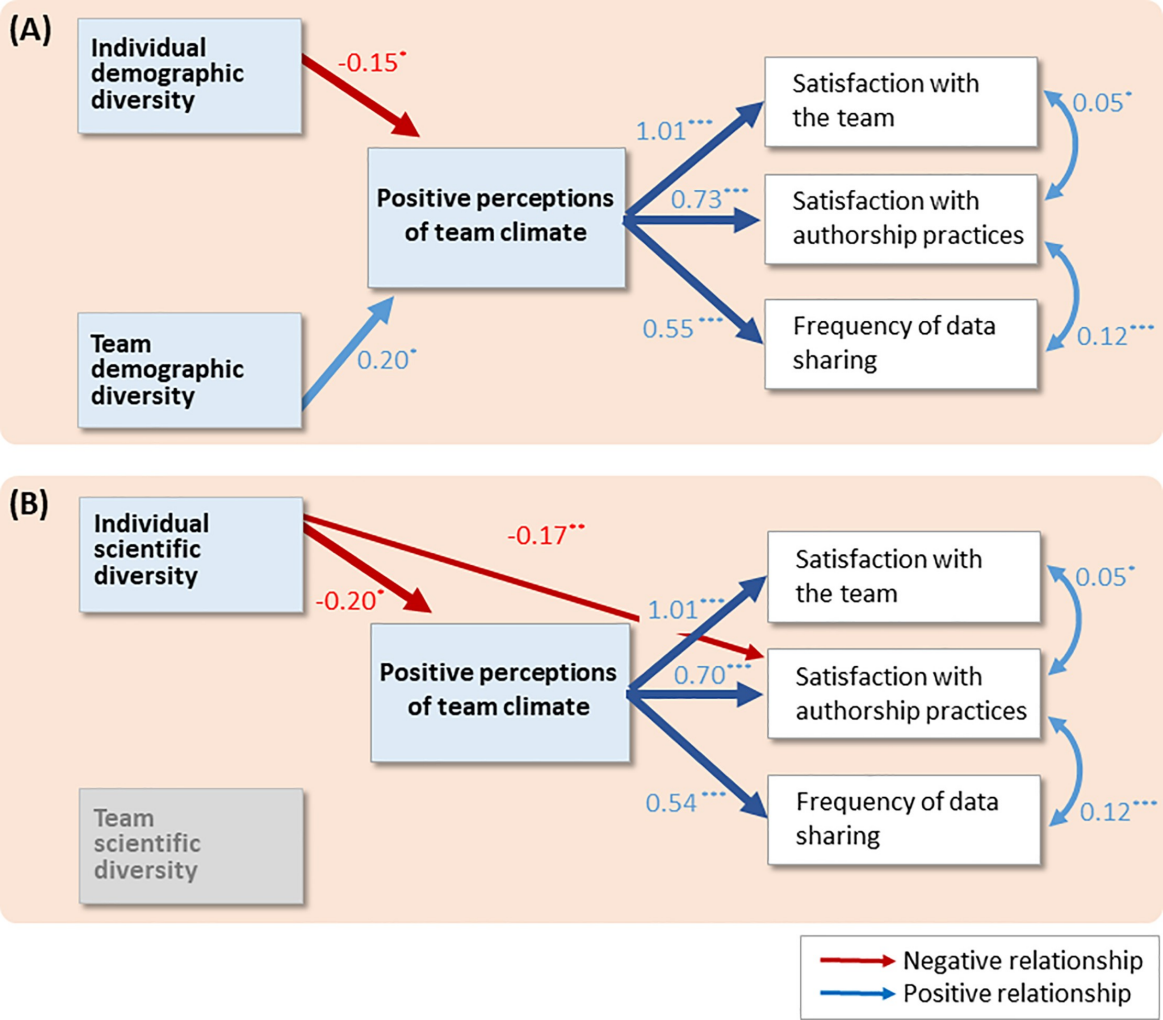
Path analysis results. Individual and team a) *demographic* diversity and b) *scientific* diversity effects on environmental science team outcomes, as mediated by climate perceptions. Numbers next to arrows indicate the coefficients, and asterisks show level of statistical significance (* *p* < .05, ** *p* < .01, *** *p* < .001); only significant paths are shown.

The first model (Fig 4A) examined the effects of *demographic diversity* on outcomes at the individual and team levels. Testing H_1_ and H_2_ for demographic diversity, we found that diversity composites were not directly related to our outcomes. However, individual demographic diversity was associated with more negative perceptions of team climate, and team demographic diversity was associated with more positive perceptions of team climate. Further, tests of indirect effects indicated that team climate perceptions mediated the relationship between individual demographic diversity and all three outcomes. Team climate also mediated the relationship between team demographic diversity and all three outcomes, but with opposite effects (See Table 4). Specifically, participants with more underrepresented demographic characteristics (e.g., women who are Black, gay men not born in the US) perceived their team climate to be more negative, which was associated with lower satisfaction with the team and more negative perceptions of authorship and data sharing on their teams. In contrast, participants on more demographically diverse teams perceived team climate to be more positive, which was associated with their greater satisfaction with the team and more positive perceptions of authorship and data sharing on their teams.

**Table 4:** Indirect effects of individual demographic diversity, team demographic diversity, individual scientific diversity, and team scientific diversity on satisfaction, authorship, and data sharing through the team climate mediator.

	Mediator: Team Climate		
	Satisfaction	Authorship	Data Sharing
	Estimate (95% CI)	Estimate (95% CI)	Estimate (95% CI)
Individual Demographic Diversity	-.15* (-.24, -.05)	-.11* (-.18, -.04)	-.08* (-.13, -.02)
Team Demographic Diversity	.21* (.06, .35)	.15* (.04, .25)	.11 (.03, .19)
Individual Scientific Diversity	-.20* (-.31, -.09)	-.14* (-.22, -.06)	-.11* (-.17, -.04)
Team Scientific Diversity	.06 (-.07, .18)	.04 (-.05, .13)	.03 (-.04, .10)

*Note*. 5,000 bootstrap resamples were used. Estimate = Unstandardized estimate of indirect effect.

* = Upper and lower 95% confidence interval does not contain 0.

### Scientific diversity and team climate

The second model (Fig 4B) examined the role of *scientific diversity* at the individual and team levels. Testing H_1_ and H_2_ for scientific diversity, we found that individual scientific diversity was directly related to satisfaction with authorship practices. As with individual demographic diversity, individuals who contributed more scientific diversity to their teams perceived their team climate more negatively. However, unlike team demographic diversity, team scientific diversity was unrelated to team climate. Tests of indirect effects indicated that team climate mediated the relationship between individual scientific diversity and all three outcomes. However, team climate did not mediate the relationship between team scientific diversity and any outcome. Thus, individuals with more underrepresented or low status scientific characteristics perceived their team climate more negatively, which was associated with their lower satisfaction with the team and more negative authorship and data sharing perceptions. In contrast, team scientific diversity was not related to team climate or any of the outcomes examined.

## Discussion

Our findings indicate that positive perceptions of team climate are associated with satisfaction with teams, as well as perceptions that authorship practices are fair and that data are shared openly within teams. However, individuals with more dimensions of demographic or scientific diversity (e.g., women, LGBTQ team members, early-career scientists) perceived team climate to be more negative than their more represented counterparts, and as a result they reported less satisfaction with their teams, team authorship practices, and the frequency of team data sharing.

These findings support our hypotheses and suggest that efforts to maximize the benefits and minimize the challenges of diverse science teams should take into account the mediating effects of team climate. In accordance with our first hypothesis, we found that those who contributed more demographic or scientific diversity tended to perceive climate less positively than those who did not contribute as much diversity. As predicted by our second hypothesis, one of the factors related to positive climate perceptions was team *demographic* diversity, although team *scientific* diversity did not have this effect. Thus, although our results support ongoing efforts within the scientific community to incorporate individuals who can contribute diversity to scientific teams, we add the important caveat that it is critical to provide these individuals with adequate support and recognition. Moreover, in order to promote positive team outcomes, greater attention needs to be directed at understanding the range of factors that influence the climate of science teams.

As predicted by H_1_ and H_2_, perceptions of team climate on diverse science teams may drive outcomes such as satisfaction with teams, satisfaction with authorship practices, and frequency of data sharing. It makes sense that these outcomes can be improved by addressing the three dimensions of climate examined in this study: procedural justice, collaboration, and inclusion. Having clear, openly-discussed, and collaboratively developed team policies and practices is likely to promote data sharing and encourage fair credit allocation related to authorship [12,28,31]. In addition, fair and transparent policies and procedures are likely to alleviate power imbalances that can diminish satisfaction with teams [12]. The importance of promoting positive climate also accords with the finding that diversity can have varied effects on team outcomes, and what matters is whether organizations support diversity by recognizing the contributions of all individuals through fair processes and rewards [32].

We found that perceptions of the climate on teams with greater demographic diversity were more positive than on less demographically diverse teams. These positive effects of demographic diversity are aligned with previous research indicating that diversity can have a number of beneficial effects on team outcomes [5,33]. Demographic diversity might improve team climate because team members from traditionally underrepresented groups may be particularly likely to identify concerns about power dynamics and unfair or exclusive practices on these teams [34,35]. By doing so, they could help to prevent and alleviate policies and practices that damage team climate, but they may feel frustrated or burdened with the need to be the individuals performing these extra duties.

Although demographic diversity is generally beneficial for teams, the outcomes are less positive for the individuals who contribute diversity. The less positive outcomes for these individuals may be the result of “token effects,” which occur when group members experience stresses such as performance pressure and social isolation because they have characteristics that are unique within their groups [27,36,37]. The somewhat counter-intuitive difference between group-level and individual-level results for teams with greater demographic diversity might be occurring because participants who contributed diversity to the teams we studied made up a low proportion of their teams. Therefore, their negative perceptions did not overwhelm the overall positive perceptions associated with diverse teams.

Although some scholars have theorized that token effects could be addressed by increasing the proportion of underrepresented individuals on teams [36], other research suggests that the problems experienced by token team members are related not just to low numbers but also to low status [12,38]. This accords with our findings, insofar as those who are from scientifically or demographically underrepresented groups (e.g., having early career status; being on the team for less than half the project duration; identifying sex as female; identifying race as Black, Latinx, or American Indian) are also likely to have comparatively low status on scientific teams. Thus, in addition to recruiting more individuals from underrepresented groups to science, it is important to take additional steps at the team and institutional levels to support and value the contributions of all team members [27,39,40]. Over the long term, changes to institutional cultures (i.e., the basic underlying assumptions and espoused values) in which science teams operate could help improve climate and facilitate the development of more inclusive practices [41].

Our research moves beyond previous studies in three important ways. First, very little previous work investigates the role of climate in science teams, and none of this research investigates the effects of climate on team practices like authorship and data sharing. Second, whereas previous studies have focused on single dimensions of diversity (primarily gender or race), our study answers recent calls to examine multiple dimensions of both demographic and scientific diversity [42]. Third, we examined diversity in the composition of teams at both the individual and team level (albeit aggregated from individual reports). Our findings indicate that investigations into divergences between individual and team level diversity are very important in order to promote the interests of underrepresented groups in science.

Our findings also suggest that we should reframe the current dialogue surrounding science teams and diversity. This conversation should focus less on whether diverse teams are good for team outcomes (which appears to depend on the outcomes and the dimensions of diversity) and more on the factors that contribute to positive outcomes both for diverse teams and for individual team members. We found that team climate perceptions are one of the important factors related to positive or negative outcomes on science teams. Therefore, science teams will benefit from additional research on steps to improve team climate for all members on science teams, especially those who are underrepresented or marginalized.

## Supporting information

S1 Table Diversity composites. (DOCX)

S2 Table Team climate measures. (DOCX)

## References

[1] Wuchty S, Jones BF, Uzzi B. The Increasing Dominance of Teams in Production of Knowledge. Science. 2007;316: 1036–1039. 10.1126/science.1136099 17431139

[2] Jones BF, Wuchty S, Uzzi B. Multi-University Research Teams: Shifting Impact, Geography, and Stratification in Science. Sci New Ser. 2008;322: 1259–1262. 10.1126/science.1158357 18845711

[3] Council NR. Enhancing the Effectiveness of Team Science [Internet]. 2015 10.17226/19007 26247083

[4] Eigenbrode SD, O’rourke M, Wulfhorst JD, Althoff DM, Goldberg CS, Merrill K, et al Employing Philosophical Dialogue in Collaborative Science. BioScience. 2007;57: 55–64. 10.1641/B570109

[5] Hall KL, Vogel AL, Huang GC, Serrano KJ, Rice EL, Tsakraklides SP, et al The science of team science: A review of the empirical evidence and research gaps on collaboration in science. Am Psychol. 2018;73: 532–548. 10.1037/amp0000319 29792466

[6] Bumpus N. Moving toward inclusion. Sci AAAS. 2015;350 10.1126/science.caredit.a1500273

[7] Soranno PA, Cheruvelil KS, Elliott KC, Montgomery GM. It’s Good to Share: Why Environmental Scientists’ Ethics Are Out of Date. BioScience. 2015;65: 69–73. 10.1093/biosci/biu169 26955073 PMC4776715

[8] Sarsons H. Recognition for Group Work: Gender Differences in Academia. Am Econ Rev. 2017;107: 141–145. 10.1257/aer.p20171126

[9] West JD, Jacquet J, King MM, Correll SJ, Bergstrom CT. The Role of Gender in Scholarly Authorship. PLOS ONE. 2013;8: e66212 10.1371/journal.pone.0066212 23894278 PMC3718784

[10] MacMynowski DP. Pausing at the Brink of Interdisciplinarity: Power and Knowledge at the Meeting of Social and Biophysical Science. Ecol Soc. 2007;12 Available: https://www.jstor.org/stable/26267854

[11] Norris PE, O’Rourke M, Mayer AS, Halvorsen KE. Managing the wicked problem of transdisciplinary team formation in socio-ecological systems. Landsc Urban Plan. 2016;154: 115–122. 10.1016/j.landurbplan.2016.01.008

[12] Settles IH, Brassel ST, Montgomery GM, Elliott KC, Soranno PA, Cheruvelil KS. Missing the Mark: A New Form of Honorary Authorship Motivated by Desires for Inclusion. Innov High Educ. 2018;43: 303–319. 10.1007/s10755-018-9429-z

[13] Thayer AL, Petruzzelli A, McClurg CE. Addressing the paradox of the team innovation process: A review and practical considerations. Am Psychol. 2018;73: 363–375. 10.1037/amp0000310 29792454

[14] Buttner EH, Lowe KB. Addressing internal stakeholders’ concerns: The interactive effect of perceived pay equity and diversity climate on turnover intentions. J Bus Ethics. 2017;143: 621–633. 10.1007/s10551-015-2795-x

[15] Guenter H, Gardner WL, Davis McCauley K, Randolph-Seng B, Prabhu VP. Shared Authentic Leadership in Research Teams: Testing a Multiple Mediation Model. Small Group Res. 2017;48: 719–765. 10.1177/1046496417732403 29187779 PMC5682574

[16] McKAY PF, Avery DR, Morris MA. Mean Racial-Ethnic Differences in Employee Sales Performance: The Moderating Role of Diversity Climate. Pers Psychol. 2008;61: 349–374. 10.1111/j.1744-6570.2008.00116.x

[17] Fransen K, Delvaux E, Mesquita B, Van Puyenbroeck S. The Emergence of Shared Leadership in Newly Formed Teams With an Initial Structure of Vertical Leadership: A Longitudinal Analysis. J Appl Behav Sci. 2018;54: 140–170. 10.1177/0021886318756359

[18] Whitman DS, Caleo S, Carpenter NC, Horner MT, Bernerth JB. Fairness at the collective level: A meta-analytic examination of the consequences and boundary conditions of organizational justice climate. J Appl Psychol. 2012;97: 776–791. 10.1037/a0028021 22486364

[19] Li C-R, Lin C-J, Tien Y-H, Chen C-M. A Multilevel Model of Team Cultural Diversity and Creativity: The Role of Climate for Inclusion. J Creat Behav. 2017;51: 163–179. 10.1002/jocb.93

[20] Carson JB, Tesluk PE, Marrone JA. Shared Leadership in Teams: An Investigation of Antecedent Conditions and Performance. Acad Manage J. 2007;50: 1217–1234. 10.2307/20159921

[21] Colquitt JA. On the dimensionality of organizational justice: A construct validation of a measure. J Appl Psychol. 2001;86: 386–400. 11419799 10.1037/0021-9010.86.3.386

[22] Pugh SD, Dietz J, Brief AP, Wiley JW. Looking inside and out: The impact of employee and community demographic composition on organizational diversity climate. J Appl Psychol. 2008;93: 1422–1428. 10.1037/a0012696 19025258

[23] Settles IH, Brassel ST. Science Teams’ Effective Practices. Interuniversity Consortium for Political and Social Research. 2019. 10.3886/E105622V1 Accessed: 6/26/19

[24] U.S. Census Bureau I of ES National Center for Education Statistics. Population Estimates [Internet]. 2016. Available: https://www.census.gov/quickfacts/fact/table/US/RHI105210#viewtop

[25] U.S. Department of Education, Institute of Education Sciences, National Center for Education Statistics. Full-time faculty in degree-granting postsecondary institutions, by race/ethnicity, sex, and academic rank: Fall 2011, fall 2013, and fall 2015 [Internet]. 2016. Available: https://nces.ed.gov/programs/digest/d16/tables/dt16_315.20.asp

[26] Ghavami N, Peplau LA. An Intersectional Analysis of Gender and Ethnic Stereotypes: Testing Three Hypotheses. Psychol Women Q. 2013;37: 113–127. 10.1177/0361684312464203

[27] Settles IH, Buchanan NT, Dotson K. Scrutinized but not recognized:(In) visibility and hypervisibility experiences of faculty of color. J Vocat Behav. 2018;In press. 10.1016/j.jvb.2017.11.001

[28] Elliott KC, Settles IH, Montgomery GM, Brassel ST, Cheruvelil KS, Soranno PA. Honorary Authorship Practices in Environmental Science Teams: Structural and Cultural Factors and Solutions. Account Res. 2017;24: 80–98. 10.1080/08989621.2016.1251320 27797590

[29] Lleras C. Path Analysis. Encycl Soc Meas. 2005;3: 25–30.

[30] Muthen LK, Muthen BO. MPlus Version 8 Los Angeles, CA: Muthen & Muthen; 1998.

[31] Cheruvelil KS, Soranno PA, Weathers KC, Hanson PC, Goring SJ, Filstrup CT, et al Creating and maintaining high-performing collaborative research teams: the importance of diversity and interpersonal skills. Front Ecol Environ. 2014;12: 31–38. 10.1890/130001

[32] Avery DR, McKay PF. Doing Diversity Right: An Empirically Based Approach to Effective Diversity Management International Review of Industrial and Organizational Psychology 2010. Wiley-Blackwell; 2010 pp. 227–252. 10.1002/9780470661628.ch6

[33] Woolley AW, Chabris CF, Pentland A, Hashmi N, Malone TW. Evidence for a collective intelligence factor in the performance of human groups. Science. 2010;330: 686–688. 10.1126/science.1193147 20929725

[34] Harding S. Standpoint Theories: Productively Controversial. Hypatia. 2009;24: 192–200. 10.1111/j.1527-2001.2009.01067.x

[35] Wylie, Alison R. Why Standpoint Matters In: Figeroa, Robert, Harding, Sandra G., editors. Science and Other Cultures: Issues in Philosophies of Science and Technology. Psychology Press; 2003 pp. 26–48.

[36] Kanter RM. Men and women of the corporation. New York: Basic Books; 1977.

[37] Seyranian V, Atuel H, Crano WD. Dimensions of Majority and Minority Groups. Group Process Intergroup Relat. 2008;11: 21–37. 10.1177/1368430207084843

[38] Williams CL. The Glass Escalator, Revisited: Gender Inequality in Neoliberal Times, SWS Feminist Lecturer. Gend Soc. 2013;27: 609–629. 10.1177/0891243213490232

[39] Shore LM, Randel AE, Chung BG, Dean MA, Holcombe Ehrhart K, Singh G. Inclusion and Diversity in Work Groups: A Review and Model for Future Research. J Manag. 2011;37: 1262–1289. 10.1177/0149206310385943

[40] Turner C, Gonzalez J, Wood J. Faculty of color in academe: What 20 years of literature tells us. J Divers High Educ. 2008;1: 139–168.

[41] Ostroff C, Kinicki A J, Muhammad RS. Chapter 24: Organizational culture and climate. Handbook of psychology, industrial and organizational psychology (2nd edition). 2nd ed. 2012.

[42] Shore LM, Chung-Herrera BG, Dean MA, Ehrhart KH, Jung DI, Randel AE, et al Diversity in organizations: Where are we now and where are we going? Hum Resour Manag Rev. 2009;19: 117–133. 10.1016/j.hrmr.2008.10.004

