# Supplementary material for: Team climate mediates the effect of diversity on environmental science team satisfaction and data sharing
Source: PLoS One. 2019 Jul 18;14(7):e0219196. doi: 10.1371/journal.pone.0219196 (PMC6638994; doi:10.1371/journal.pone.0219196)
Supplement: S1 Table — (DOCX) [file pone.0219196.s001.docx]

**Supplementary Materials**

| **S1 Table.** Diversity Composites |
| --- |
|  |
| *Individual Demographic Diversity* |
| Count variable representing the number of dimensions along which the participant contributes to demographic diversity on the team through their sex, gender, sexual orientation, race, and/or nationality: |
| - Sex: female = 1, male =0 |
| - Gender: trans or genderqueer = 1, cisgender =0 |
| - Sexual orientation: LGBQ =1, heterosexual/straight = 0 |
| - Race: Black, Latinx, Middle Eastern, Native American = 1, Asian, Asian American, Pacific Islander, White =0 |
| - Nationality: not from the U.S. = 1, from the U.S. =0 |
| The variable ranges from 0-5 and is coded such that individuals with higher scores have a greater number of the above five dimensions (e.g., a cisgender *bisexual* White *woman* from *Ireland* would receive a 3). |
|  |
| *Team Demographic Diversity* |
| Mean of four standardized variables measuring the proportion of team members who are: |
| - Women |
| - Racial minorities (Black, Latinx, Middle Eastern, or Native American) |
| - Sexual minorities (LGBQ) |
| - International (born outside the U.S.) |
| The variable is coded such that higher scores indicate that the team has a greater proportion of team members falling into these four dimensions. |
|  |
| *Individual Scientific Diversity* |
| Count variable representing the number of dimensions along which the participant contributes to scientific diversity on their team through their discipline, career status, and/or duration on project: |
| - Discipline: non-natural science environmental science = 1, natural science environmental science = 0 |
| - Career status: untenured/fixed-term assistant or associate professor/scientist and below = 1, tenured associate professors, fixed-term or tenured full professors, and senior scientists =0 |
| - Duration on project: less than half of the project duration or for a very short time/just started = 1, half of the project duration or more = 0 |
| The variable ranges from 0-3 and is coded such that individuals with higher scores have a greater number of the above three dimensions (e.g., a *graduate student* in the *humanities* who has been on the project for its entirety would receive a 2). |
|  |
| *Team Scientific Diversity* |
| Mean of three standardized variables measuring the proportion of team members who are: |
| - Outside natural science environmental science |
| - Earlier career status (not PIs or co-PIs) |
| - New to the team |
| The variable is coded such that higher scores indicate that the team has a greater proportion of team members falling into these three dimensions. |
