# Supplementary material for: Team climate mediates the effect of diversity on environmental science team satisfaction and data sharing
Source: PLoS One. 2019 Jul 18;14(7):e0219196. doi: 10.1371/journal.pone.0219196 (PMC6638994; doi:10.1371/journal.pone.0219196)
Supplement: S2 Table — (DOCX) [file pone.0219196.s002.docx]

| **S2 Table.** Team Climate Measures |
| --- |
|  |
| *Procedural Justice* |
| In your NSF team, to what extent: |
| 1. Have you had the ability to influence your NSF team’s policies and/or practices related to conducting and publishing research? |
| 1. Have policies been applied consistently and equally to everyone? |
| 1. Have the policies upheld ethical and moral standards? |
| 1. Have you been able to express your views and feelings? |
|  |
| *Team Collaboration* |
| Please indicate the extent to which the following characteristics describe your NSF team. |
| 1. Everyone on this team has a chance to participate and provide input. |
| 1. My team supports everyone actively participating in decision making |
| The members of the NSF team… |
| 1. …spend time discussing our team's purpose, goals, and expectations for the project |
| 1. ...devise action plans and time schedules that allow for meeting our team's goals. |
| 1. ...recognize each other's accomplishments and hard work. |
| 1. ...give encouragement to team members who seem frustrated. |
|  |
| *Team Value of Inclusion* |
| Please indicate the extent to which the following characteristics describe your NSF team. |
| 1. Our team makes it easy for people from diverse backgrounds to fit in and be accepted. |
| 1. Team members are given research opportunities within the team without regard to their gender or their racial, religious, or cultural background. |
| 1. Team leaders demonstrate through their actions that they want a diverse team. |
| 1. Team leaders do a good job of managing people with diverse backgrounds (in terms of age, sex, race, religion, or culture). |

*Note*. Procedural Justice was measured on a 5-point Likert-type scale ranging from 1 (*not at all*) to 5 (*always*) (Colquitt, 2001). Team Collaboration and Team Value of Inclusion were both measured on a 5-point Likert-type scale ranging from 1 (*strongly disagree*) to 5 (*strongly agree*) (Carson, Tesluk, & Maronne, 2007; Pugh, Dietz, Brief, & Wiley, 2008). The correlations between all measures were significant with *p* < .001: Procedural Justice and Team Collaboration, *r* = .66; Procedural Justice and Team Value of Inclusion, *r* = .70; Team Collaboration and Team Value of Inclusion, *r* = .72.
